# Post-Pandemic Challenges: Endemic COVID-19, Vaccine Hesitancy, and Viral Resurgence

**DOI:** 10.3390/tropicalmed11040097

**Published:** 2026-04-05

**Authors:** Constantinos Tsioutis, Marcin Piotr Walkowiak

**Affiliations:** 1 School of Medicine, European University Cyprus, 6 Diogenes Str., Nicosia 2404, Cyprus; 2 Department of Preventive Medicine, Poznan University of Medical Sciences, Święcickiego Str. 6, 60-781 Poznan, Poland; marcinwalkowiak@ump.edu.pl

**Keywords:** vaccine hesitancy, COVID-19, epidemiology, endemic viruses, natural immunity, post-pandemic

Paraphrasing T. S. Eliot, the COVID-19 pandemic ended not with a bang but with a whimper. The conclusion of the COVID-19 global health emergency did not signal the disappearance of SARS-CoV-2; rather, it marked the beginning of a challenging transition toward endemicity. In hindsight, this development is not surprising: a close relative of the virus responsible for the Spanish Flu—the A(H1N1) influenza virus—still causes annual seasonal epidemics. This Special Issue explores the “new normal,” characterized by the persistent circulation of the SARS-CoV-2 virus, the sociological challenges of vaccine hesitancy, and the resurgence of other respiratory pathogens that were suppressed during periods of restriction. The published studies in this Special Issue provide critical insights into clinical outcomes, epidemiological patterns, and the behavioral factors that define this post-pandemic era.

A central focus of the post-pandemic era is the evolving clinical profile of COVID-19. Todorov et al. demonstrated the prognostic utility of the age-adjusted Charlson Comorbidity Index (CCI), demonstrating its efficacy in predicting disease severity and mortality in a cohort of 373 hospitalized patients with underlying comorbidities, particularly diabetes mellitus and hypertension [1]. Their findings emphasize that even as the virus evolves into milder forms, pre-existing chronic conditions, particularly with a CCI above 5, remain a significant determinant of severe disease and worse outcomes. Similarly, a retrospective observational study of 894,326 COVID-19 patients in Colombia identified a stronger association of death with lack of vaccination, older age, greater bodyweight, and number of comorbidities [2]. Complementing these findings, a population-based prospective study from Spain analyzed the association between vitamin D levels and COVID-19 reinfection, identifying higher risk in cases with low (<30 ng/mL) levels [3].

The shift towards endemicity also requires improved tools for surveillance and diagnosis. Researchers from Brazil analyzed a large telemedicine database to develop a diagnostic prediction model, identifying specific symptoms as key predictors that can help clinicians to distinguish COVID-19 from other influenza-like illnesses in a remote setting [4]. Among the 2548 patients included in the analysis, tiredness and fatigue were more prevalent in COVID-19 cases, while rhinorrhea, sneezing and burning nose, ocular symptoms, abdominal pain, rhinosinusopathy, and wheezing/bronchospasm were more frequent in H1N1 controls. This model highlights the practical value of digital health for remote triage and differentiation of respiratory infections in resource-limited or post-peak settings.

A systematic review by Farias et al. investigated the epidemiology of meningitis during and after the COVID-19 pandemic, highlighting a possible post-pandemic resurgence of various etiologic agents based on the reported case numbers [5]. Despite the heterogeneity of the included studies and possible publication bias, particularly from developing areas of the world, the global impact of COVID-19 on emerging and re-emerging diseases warrants vigilance and robust surveillance.

Three studies in Asia analyze geographical and temporal perspectives on viral dynamics. Hengkrawit et al. compared the clinical characteristics of 1084 hospitalized patients in a tertiary care hospital in Thailand across the Alpha, Delta, and Omicron waves, noting that while Omicron was generally milder in terms of disease severity and mortality, 75% of patients who died were over 65 years of age [6]. Wang et al. conducted a large-scale case–control study in China during the Omicron wave, aiming to identify risk factors for severe and persistent symptoms (exceeding 21 days after COVID-19 onset) [7]. Female sex, history of allergies, overweight/obesity (BMI ≥ 24), coronary artery disease, stroke, weekly coffee consumption, and having the presence at least one comorbidity were also independent risk factors for persistent symptoms, while female sex, history of allergies, and having three or more comorbidities were associated with severe symptoms. The “pandemic-to-epidemic” transition was examined by a retrospective epidemiological analysis of cluster outbreaks in Shanghai [8]. By analyzing 67 clusters, the study highlighted that workplaces are settings of larger-scale transmission, and that as broad community interventions are phased out, targeting household and workplace clusters remains the most effective strategy to prevent surges.

The COVID-19 pandemic both revealed and, in some cases, amplified vaccine hesitancy, which continues to challenge efforts toward stable endemicity. A study from Poland investigated the underreporting of adverse events following immunization (AEFIs) by performing a cross-sectional survey among healthcare professionals (*n* = 1063) and medical students (*n* = 1506), comparing their responses to national official reports [9]. The authors detected discrepancies between self-reported symptoms and official records, implying significant differences in notification rates, an observation that can inadvertently cause mistrust and hesitancy, thus calling for robust pharmacovigilance systems. To this end, a cross-sectional study in Morocco investigating the knowledge of 200 nurses in regard to novel COVID-19 vaccines [10] highlighted significant knowledge gaps and the need for continuous education and training in pharmacovigilance issues.

In conclusion, this collection of research underscores that the post-pandemic phase is not a return to the status quo, but a period of active adaptation. The lessons learned regarding clinical risk, the importance of vaccine trust, and the utility of digital surveillance will be vital in preparing for future viral resurgences and ensuring the resilience of global health systems.

## Data Availability

Not applicable.
